# Crystal structure of *catena*-poly[[[tri­aqua­strontium]-di-μ_2_-glycinato] dibromide]

**DOI:** 10.1107/S2056989015012219

**Published:** 2015-06-30

**Authors:** Palanisamy Revathi, Thangavelu Balakrishnan, Kandasamy Ramamurthi, Subbiah Thamotharan

**Affiliations:** a Crystal Growth Laboratory, PG and Research Department of Physics, Periyar EVR College (Autonomous), Tiruchirappalli 620 023, India; b Crystal Growth and Thin Film Laboratory, Department of Physics and Nanotechnology, SRM University, Kattankulathur 603 203, India; c Biomolecular Crystallography Laboratory, Department of Bioinformatics, School of Chemical and Biotechnology, SASTRA University, Thanjavur 613 401, India

**Keywords:** crystal structure, glycine, strontium, N/O—H⋯Br/O hydrogen bonds

## Abstract

In the title coordination polymer, {[Sr(C_2_H_5_NO_2_)_2_(H_2_O)_3_]Br_2_}_*n*_, the Sr^2+^ ion and one of the water mol­ecules are located on twofold rotation axes. The alkaline earth ion is nine-coordinated by three water O atoms and six O atoms of the carboxyl­ate groups of four glycine ligands, two in a chelating mode and two in a monodentate mode. The glycine mol­ecule exists in a zwitterionic form and bridges the cations into chains parallel to [001]. The Br^−^ counter-anions are located between the chains. Inter­molecular hydrogen bonds are formed between the amino and carboxyl­ate groups of neighbouring glycine ligands, generating a head-to-tail sequence. Adjacent head-to-tail sequences are further inter­connected by inter­molecular N—H⋯Br hydrogen-bonding inter­actions into sheets parallel to (100). O—H⋯Br and O—H⋯O hydrogen bonds involving the coordinating water mol­ecules are also present, consolidating the three-dimensional hydrogen-bonding network.

## Abstract 2

The characteristic structural feature of the title compound is the formation of cationic chains extending parallel to [001], with the Br^−^ counter-anions located in between. Inter­molecular N—H⋯O, N—H⋯Br, O—H⋯O and O—H⋯Br hydrogen bonds stabilize the structure.

## Chemical context   

Research in the field of coordination polymers has undergone rapid development in recent years due to their inter­esting structures and their wide range of applications as functional materials (Lyhs *et al.*, 2012 ▸). One of the simplest amino acids is glycine and some glycine–metal complexes have been reported previously (Fleck *et al.*, 2006 ▸ and references therein). The crystal structures of strontium combined with anions of amino acids are rare. As part of our ongoing investigations of the crystal and mol­ecular structures of a series of metal complexes derived from amino acids (Sathiskumar *et al.*, 2015**a* ▸,b* ▸; Balakrishnan *et al.*, 2013 ▸), we report here the crystal structure of a polymeric strontium–glycine complex, {[Sr(C_2_H_5_NO_2_)_2_(H_2_O)_3_]Br_2_}_*n*_, (I).
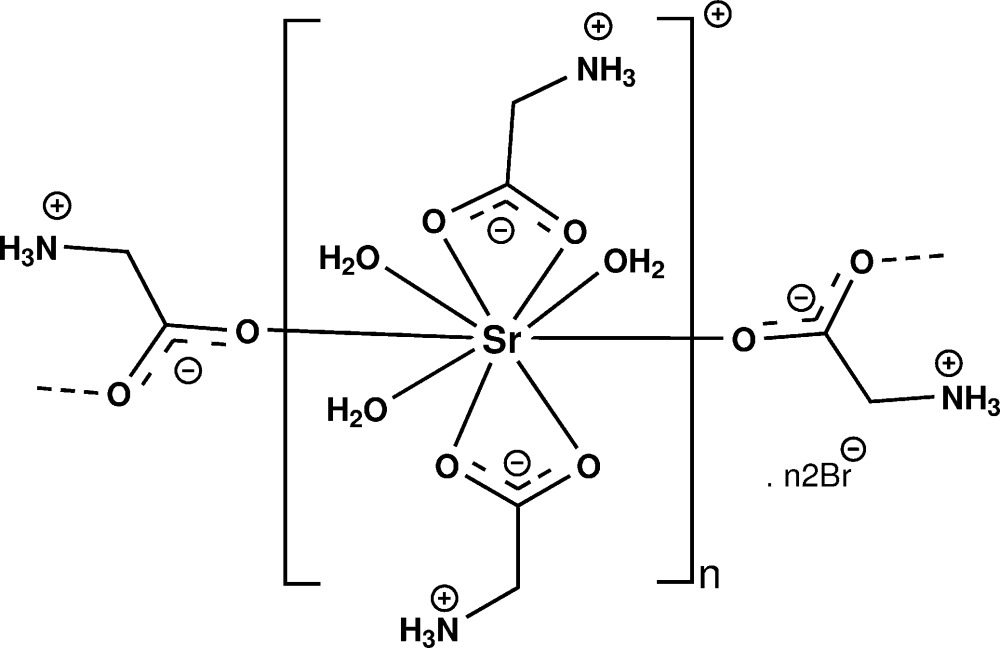


## Structural commentary   

**Figure 1:**
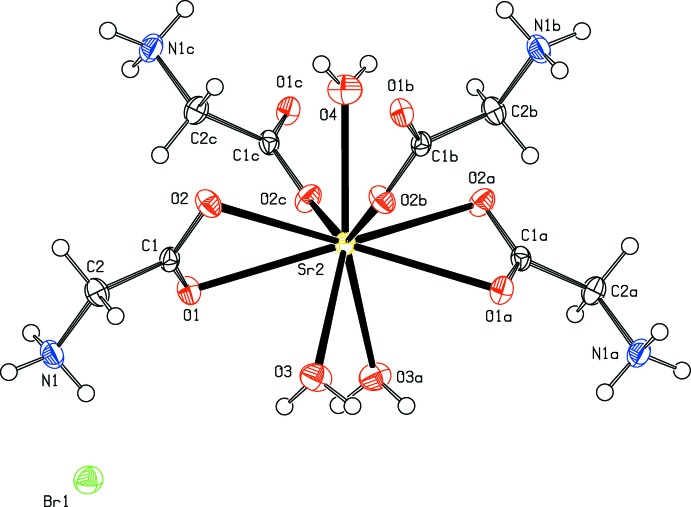
The coordination environment of Sr^2+^ in the crystal structure of (I). Displacement ellipsoids are drawn at the 40% probability level. [Symmetry codes: (*a*) −*x*, *y*, 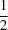 − *z*; (*b*) −*x*, 1 − *y*, 1 − *z*; (*c*) *x*, 1 − *y*, −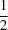 + *z*].

**Figure 2:**
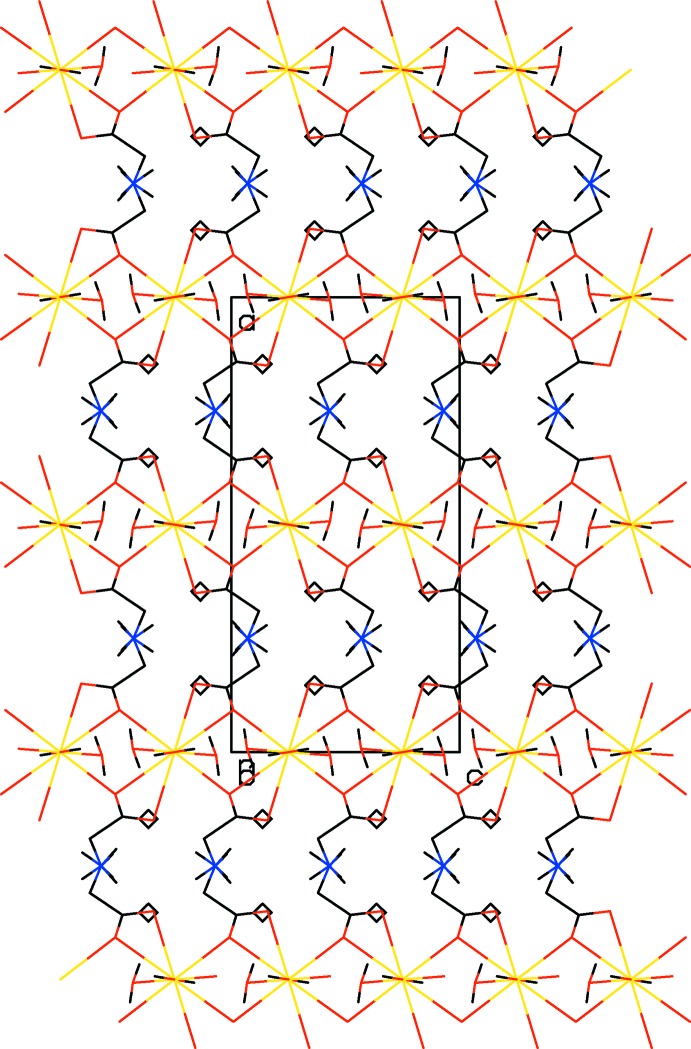
The crystal packing of (I) projected along [010]. H atoms have been omitted for clarity.

The asymmetric unit of (I) contains one Sr^2+^ ion, one glycine ligand, one and a half water mol­ecules and one bromide anion (Fig. 1 ▸). The Sr^2+^ cation and one of the water mol­ecules (O4) are located on special positions with site symmetry 2. The bond lengths involving the carboxyl­ate atoms and the proton­ation of the amino group reveal a zwitterionic form for the glycine ligand in (I). The Sr^2+^ ion is nine-coordinated by three oxygen atoms [Sr—O = 2.526 (4)–2.661 (2) Å] of water mol­ecules and six carboxyl­ate oxygen atoms of four glycine ligands [Sr—O = 2.605 (2)–2.703 (2) Å]. The glycine ligands coordinate each cation in a bis-bidentate and bis-monodentate way and simultaneously bridge two alkaline earth cations. As shown in Fig. 2 ▸, this coordination mode leads to the formation of polymeric chains running parallel to [001]. Adjacent Sr^2+^ ions are separated by 4.3497 (3) Å within a chain and the shortest Sr⋯Sr distance between neighbouring chains is 9.4960 (3) Å.

## Supra­molecular features   

**Figure 3:**
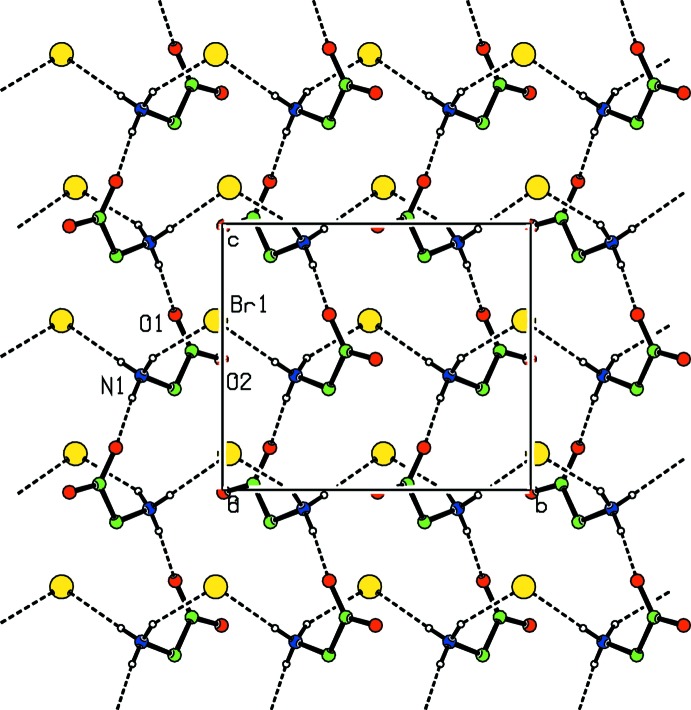
Zwitterionic glycine mol­ecules are inter­connected by inter­molecular N—H⋯O and N—H⋯Br hydrogen bonds into (100) sheets.

**Figure 4:**
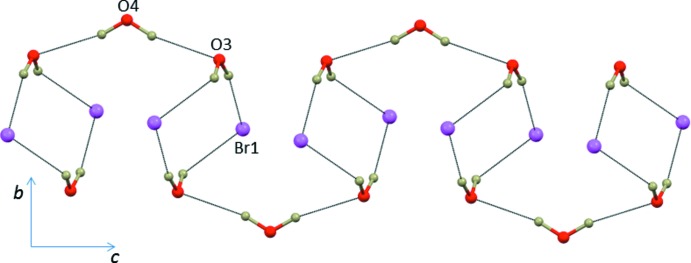
Cyclic dibromide motifs are inter­connected by inter­molecular O—H⋯O inter­actions.

The crystal structure of (I) contains an intricate network of inter­molecular N—H⋯O, N—H⋯Br, O—H⋯O and O—H⋯Br hydrogen bonds (Table 1 ▸). The protonated N atom of the glycine mol­ecule is capable of forming three hydrogen-bonding inter­actions. One of them is the characteristic head-to-tail sequence in which amino acids are self-assembled through their amino and carboxyl­ate groups (Sharma *et al.*, 2006 ▸; Selvaraj *et al.*, 2007 ▸; Balakrishnan *et al.*, 2013 ▸). In (I), the zwitterionic glycine mol­ecules are arranged in linear arrays that run parallel to the [110] direction (Fig. 3 ▸), and adjacent glycine mol­ecules are inter­connected by an inter­molecular N1—H1*A*⋯O1 hydrogen bond. This inter­action can be described as a head-to-tail sequence having a *C*(5) graph-set motif (Bernstein *et al.*, 1995 ▸). In each array, the Br^−^ counter anions bridge neighbouring glycines. Taken together, these three inter­actions form a hydrogen-bonded sheet extending parallel to (100). One of the water mol­ecules (O3) acts as a donor for two different Br^−^ anions. These inter­molecular O—H⋯Br inter­actions result in a cyclic dibromide motif as observed in the crystal structure of *N*,*N*′-dibenzyl-*N*,*N*,*N*′,*N*′-tetra­methyl­ethylenedi­ammonium dibromide dihydrate (Srinivasan *et al.*, 2006 ▸). Within this motif, the distance between Br anions is 5.3398 (3) Å, and the distance between water oxygen atoms (O3⋯O3′) is 3.932 (4) Å. Adjacent cylic dibromide motifs, which are parallel to [001], are inter­connected by another water mol­ecule (O4) (Table 1 ▸ and Fig. 4 ▸).

## Synthesis and crystallization   

Crystals of (I) were grown from an aqueous solution by slow solvent evaporation at room temperature. Analytical grade reagents glycine (Merck) and strontium bromide hexa­hydrate (Sigma–Aldrich) were taken in a 2:1 molar ratio, dissolved in double-distilled water and stirred well for 4 h using a temperature-controlled magnetic stirrer to yield a homogeneous mixture. The solution was finally filtered using Whatman filter paper. The beaker containing the solution was closed with a polythene sheet with two (or) three perforations and kept in a dust-free atmosphere for slow evaporation. Single crystals were harvested after a growth period of 20 days.

## Refinement   

Crystal data, data collection and structure refinement details are summarized in Table 2 ▸. The positions of the amino and water H atoms were located from difference Fourier maps. The O3—H3*B* and O4—H4 distances of the water mol­ecules were restrained to 0.85 (2) Å. The remaining hydrogen atoms were placed in geometrically idealized positions (C—H = 0.97 Å) with *U*_iso_(H) = 1.2*U*_eq_(C) and were constrained to ride on their parent atoms.

## Supplementary Material

Crystal structure: contains datablock(s) I. DOI: 10.1107/S2056989015012219/wm5177sup1.cif

Structure factors: contains datablock(s) I. DOI: 10.1107/S2056989015012219/wm5177Isup2.hkl

CCDC reference: 1408767

Additional supporting information:  crystallographic information; 3D view; checkCIF report

## Figures and Tables

**Table 1:** Hydrogen-bond geometry (, )

*D*H*A*	*D*H	H*A*	*D* *A*	*D*H*A*
N1H1*A*O1^i^	0.88(5)	2.00(5)	2.879(4)	175(4)
N1H1*B*Br1^ii^	0.88(4)	2.58(4)	3.450(3)	179(4)
N1H1*C*Br1^iii^	0.89(4)	2.51(4)	3.321(3)	152(3)
O4H4O3^iv^	0.83(2)	2.01(2)	2.828(3)	166(5)
O3H3*A*Br1^ii^	0.84(5)	2.50(5)	3.335(3)	170(4)
O3H3*B*Br1^v^	0.84(2)	2.55(3)	3.296(3)	148(4)

Symmetry codes: (i) 
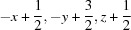
; (ii) 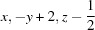; (iii) 
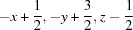
; (iv) 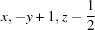; (v) 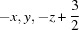.

**Table 2:** Experimental details

Crystal data	
Chemical formula	[Sr(C_2_H_5_NO_2_)_2_(H_2_O)_3_]Br_2_
*M*_r_	451.63
Crystal system, space group	Orthorhombic, *P* *b* *c* *n*
Temperature (K)	296
*a*, *b*, *c* ()	16.4198(9), 9.5438(5), 8.2402(4)
*V* (^3^)	1291.30(12)
*Z*	4
Radiation type	Mo *K*
(mm^1^)	10.38
Crystal size (mm)	0.15 0.10 0.10

Data collection	
Diffractometer	Bruker Kappa APEXII CCD
Absorption correction	Multi-scan (*SADABS*; Bruker, 1999)
*T*_min_, *T*_max_	0.251, 0.410
No. of measured, independent and observed [*I* > 2(*I*)] reflections	22178, 1564, 1244
*R*_int_	0.070
(sin /)_max_ (^1^)	0.661

Refinement	
*R*[*F* ^2^ > 2(*F* ^2^)], *wR*(*F* ^2^), *S*	0.023, 0.057, 1.14
No. of reflections	1564
No. of parameters	99
No. of restraints	2
H-atom treatment	H atoms treated by a mixture of independent and constrained refinement
_max_, _min_ (e ^3^)	0.86, 0.68

Computer programs: *APEX2* and *SAINT* (Bruker, 2004 ▸), *SIR92* (Altomare *et al.*, 1995 ▸), *SHELXL2014* (Sheldrick, 2015 ▸), *PLATON* (Spek, 2009 ▸) and *Mercury* (Macrae *et al.*, 2008 ▸).

## supplementary crystallographic information

### Crystal data

**Table:**

[Sr(C_2_H_5_NO_2_)_2_(H_2_O)_3_]Br_2_	*D*_x_ = 2.323 Mg m^−^^3^
*M**_r_* = 451.63	Mo *K*α radiation, λ = 0.71073 Å
Orthorhombic, *P**b**c**n*	Cell parameters from 6100 reflections
*a* = 16.4198 (9) Å	θ = 2.5–27.8°
*b* = 9.5438 (5) Å	µ = 10.38 mm^−^^1^
*c* = 8.2402 (4) Å	*T* = 296 K
*V* = 1291.30 (12) Å^3^	Block, colourless
*Z* = 4	0.15 × 0.10 × 0.10 mm
*F*(000) = 872	

### Data collection

**Table:**

Bruker Kappa APEXII CCD diffractometer	1244 reflections with *I* > 2σ(*I*)
Radiation source: Sealed tube	*R*_int_ = 0.070
ω and φ scan	θ_max_ = 28.0°, θ_min_ = 2.5°
Absorption correction: multi-scan (*SADABS*; Bruker, 1999)	*h* = −21→21
*T*_min_ = 0.251, *T*_max_ = 0.410	*k* = −12→12
22178 measured reflections	*l* = −9→10
1564 independent reflections	

### Refinement

**Table:**

Refinement on *F*^2^	Hydrogen site location: mixed
Least-squares matrix: full	H atoms treated by a mixture of independent and constrained refinement
*R*[*F*^2^ > 2σ(*F*^2^)] = 0.023	*w* = 1/[σ^2^(*F*_o_^2^) + (0.0169*P*)^2^ + 1.7773*P*] where *P* = (*F*_o_^2^ + 2*F*_c_^2^)/3
*wR*(*F*^2^) = 0.057	(Δ/σ)_max_ = 0.001
*S* = 1.14	Δρ_max_ = 0.86 e Å^−^^3^
1564 reflections	Δρ_min_ = −0.67 e Å^−^^3^
99 parameters	Extinction correction: *SHELXL2014* (Sheldrick 2015), Fc^*^=kFc[1+0.001xFc^2^λ^3^/sin(2θ)]^-1/4^
2 restraints	Extinction coefficient: 0.0086 (3)

### Special details

**Table:**

Geometry. All e.s.d.'s (except the e.s.d. in the dihedral angle between two l.s. planes) are estimated using the full covariance matrix. The cell e.s.d.'s are taken into account individually in the estimation of e.s.d.'s in distances, angles and torsion angles; correlations between e.s.d.'s in cell parameters are only used when they are defined by crystal symmetry. An approximate (isotropic) treatment of cell e.s.d.'s is used for estimating e.s.d.'s involving l.s. planes.

### Fractional atomic coordinates and isotropic or equivalent isotropic displacement parameters (Å^2^)

**Table:**

	*x*	*y*	*z*	*U*_iso_*/*U*_eq_	
C1	0.14184 (17)	0.5997 (3)	0.4781 (4)	0.0178 (6)	
C2	0.1901 (2)	0.6557 (3)	0.6205 (4)	0.0232 (7)	
H2A	0.1529	0.6963	0.6989	0.028*	
H2B	0.2183	0.5788	0.6729	0.028*	
N1	0.2500 (2)	0.7627 (3)	0.5708 (4)	0.0263 (6)	
O1	0.15044 (13)	0.6537 (2)	0.3416 (2)	0.0224 (5)	
O2	0.09257 (13)	0.5034 (2)	0.5090 (3)	0.0251 (5)	
O3	−0.00732 (17)	0.8029 (3)	0.4322 (3)	0.0308 (6)	
O4	0.0000	0.3083 (4)	0.2500	0.0331 (8)	
Br1	0.14700 (2)	0.97766 (4)	0.86395 (4)	0.02908 (12)	
Sr2	0.0000	0.57306 (4)	0.2500	0.01637 (12)	
H1A	0.279 (3)	0.793 (5)	0.654 (6)	0.062 (15)*	
H1B	0.224 (2)	0.830 (4)	0.520 (5)	0.046 (13)*	
H1C	0.287 (3)	0.726 (4)	0.505 (5)	0.044 (12)*	
H4	−0.007 (3)	0.264 (4)	0.164 (4)	0.064 (15)*	
H3A	0.033 (3)	0.853 (5)	0.404 (5)	0.059 (15)*	
H3B	−0.0497 (19)	0.852 (4)	0.444 (6)	0.067 (16)*	

### Atomic displacement parameters (Å^2^)

**Table:**

	*U*^11^	*U*^22^	*U*^33^	*U*^12^	*U*^13^	*U*^23^
C1	0.0131 (14)	0.0216 (15)	0.0186 (14)	0.0022 (11)	0.0002 (12)	−0.0025 (12)
C2	0.0231 (17)	0.0283 (18)	0.0183 (16)	−0.0036 (13)	−0.0017 (13)	−0.0028 (13)
N1	0.0224 (15)	0.0283 (17)	0.0283 (15)	−0.0035 (13)	−0.0052 (14)	−0.0055 (14)
O1	0.0204 (11)	0.0284 (12)	0.0184 (11)	−0.0046 (9)	−0.0007 (9)	0.0022 (9)
O2	0.0259 (12)	0.0277 (12)	0.0216 (11)	−0.0084 (9)	−0.0011 (9)	0.0018 (9)
O3	0.0295 (14)	0.0273 (14)	0.0356 (14)	−0.0015 (12)	0.0082 (12)	−0.0045 (11)
O4	0.044 (2)	0.031 (2)	0.0248 (19)	0.000	0.0019 (18)	0.000
Br1	0.02717 (19)	0.0276 (2)	0.0325 (2)	0.00143 (14)	0.00329 (15)	0.00078 (14)
Sr2	0.01582 (19)	0.0194 (2)	0.01391 (19)	0.000	−0.00064 (16)	0.000

### Geometric parameters (Å, º)

**Table:**

C1—O1	1.246 (4)	O2—Sr2	2.703 (2)
C1—O2	1.251 (3)	O3—Sr2	2.661 (2)
C1—C2	1.513 (4)	O3—H3A	0.84 (5)
C1—Sr2	3.004 (3)	O3—H3B	0.842 (19)
C2—N1	1.477 (4)	O4—Sr2	2.526 (4)
C2—H2A	0.9700	O4—H4	0.833 (19)
C2—H2B	0.9700	Sr2—O2^ii^	2.605 (2)
N1—H1A	0.88 (5)	Sr2—O2^i^	2.605 (2)
N1—H1B	0.88 (4)	Sr2—O3^iii^	2.661 (2)
N1—H1C	0.89 (4)	Sr2—O1^iii^	2.695 (2)
O1—Sr2	2.695 (2)	Sr2—O2^iii^	2.703 (2)
O2—Sr2^i^	2.605 (2)	Sr2—C1^iii^	3.004 (3)
			
O1—C1—O2	124.1 (3)	O1^iii^—Sr2—O1	146.83 (10)
O1—C1—C2	119.7 (3)	O4—Sr2—O2^iii^	75.77 (4)
O2—C1—C2	116.1 (3)	O2^ii^—Sr2—O2^iii^	69.96 (8)
O1—C1—Sr2	63.74 (15)	O2^i^—Sr2—O2^iii^	101.82 (7)
O2—C1—Sr2	64.13 (16)	O3^iii^—Sr2—O2^iii^	77.44 (7)
C2—C1—Sr2	157.1 (2)	O3—Sr2—O2^iii^	128.53 (7)
N1—C2—C1	112.2 (3)	O1^iii^—Sr2—O2^iii^	48.23 (6)
N1—C2—H2A	109.2	O1—Sr2—O2^iii^	143.76 (6)
C1—C2—H2A	109.2	O4—Sr2—O2	75.77 (4)
N1—C2—H2B	109.2	O2^ii^—Sr2—O2	101.82 (7)
C1—C2—H2B	109.2	O2^i^—Sr2—O2	69.96 (8)
H2A—C2—H2B	107.9	O3^iii^—Sr2—O2	128.53 (7)
C2—N1—H1A	111 (3)	O3—Sr2—O2	77.44 (7)
C2—N1—H1B	108 (3)	O1^iii^—Sr2—O2	143.76 (6)
H1A—N1—H1B	113 (4)	O1—Sr2—O2	48.23 (6)
C2—N1—H1C	111 (3)	O2^iii^—Sr2—O2	151.53 (9)
H1A—N1—H1C	104 (4)	O4—Sr2—C1^iii^	94.86 (6)
H1B—N1—H1C	109 (4)	O2^ii^—Sr2—C1^iii^	89.95 (7)
C1—O1—Sr2	91.77 (17)	O2^i^—Sr2—C1^iii^	92.77 (7)
C1—O2—Sr2^i^	137.62 (19)	O3^iii^—Sr2—C1^iii^	67.17 (8)
C1—O2—Sr2	91.27 (18)	O3—Sr2—C1^iii^	104.38 (8)
Sr2^i^—O2—Sr2	110.04 (8)	O1^iii^—Sr2—C1^iii^	24.49 (7)
Sr2—O3—H3A	106 (3)	O1—Sr2—C1^iii^	149.51 (7)
Sr2—O3—H3B	124 (3)	O2^iii^—Sr2—C1^iii^	24.60 (7)
H3A—O3—H3B	111 (4)	O2—Sr2—C1^iii^	161.97 (7)
Sr2—O4—H4	120 (3)	O4—Sr2—C1	94.86 (6)
O4—Sr2—O2^ii^	73.73 (5)	O2^ii^—Sr2—C1	92.77 (7)
O4—Sr2—O2^i^	73.73 (5)	O2^i^—Sr2—C1	89.95 (7)
O2^ii^—Sr2—O2^i^	147.46 (10)	O3^iii^—Sr2—C1	104.38 (8)
O4—Sr2—O3^iii^	145.52 (6)	O3—Sr2—C1	67.17 (8)
O2^ii^—Sr2—O3^iii^	76.97 (7)	O1^iii^—Sr2—C1	149.51 (7)
O2^i^—Sr2—O3^iii^	133.43 (8)	O1—Sr2—C1	24.49 (7)
O4—Sr2—O3	145.52 (6)	O2^iii^—Sr2—C1	161.97 (7)
O2^ii^—Sr2—O3	133.43 (8)	O2—Sr2—C1	24.60 (7)
O2^i^—Sr2—O3	76.96 (7)	C1^iii^—Sr2—C1	170.29 (11)
O3^iii^—Sr2—O3	68.96 (11)	O4—Sr2—Sr2^iv^	71.300 (10)
O4—Sr2—O1^iii^	106.59 (5)	O2^ii^—Sr2—Sr2^iv^	35.72 (5)
O2^ii^—Sr2—O1^iii^	113.67 (6)	O2^i^—Sr2—Sr2^iv^	129.21 (5)
O2^i^—Sr2—O1^iii^	76.03 (7)	O3^iii^—Sr2—Sr2^iv^	74.32 (6)
O3^iii^—Sr2—O1^iii^	69.40 (8)	O3—Sr2—Sr2^iv^	143.02 (6)
O3—Sr2—O1^iii^	83.18 (8)	O1^iii^—Sr2—Sr2^iv^	80.00 (4)
O4—Sr2—O1	106.59 (5)	O1—Sr2—Sr2^iv^	110.90 (4)
O2^ii^—Sr2—O1	76.03 (7)	O2^iii^—Sr2—Sr2^iv^	34.24 (5)
O2^i^—Sr2—O1	113.67 (6)	O2—Sr2—Sr2^iv^	131.99 (5)
O3^iii^—Sr2—O1	83.18 (8)	C1^iii^—Sr2—Sr2^iv^	55.55 (6)
O3—Sr2—O1	69.40 (8)	C1—Sr2—Sr2^iv^	128.31 (6)
			
O1—C1—C2—N1	−6.2 (4)	O1—C1—O2—Sr2^i^	−144.8 (2)
O2—C1—C2—N1	176.5 (3)	C2—C1—O2—Sr2^i^	32.3 (4)
Sr2—C1—C2—N1	−98.5 (5)	Sr2—C1—O2—Sr2^i^	−122.2 (3)
O2—C1—O1—Sr2	22.7 (3)	O1—C1—O2—Sr2	−22.6 (3)
C2—C1—O1—Sr2	−154.3 (2)	C2—C1—O2—Sr2	154.5 (2)

Symmetry codes: (i) −*x*, −*y*+1, −*z*+1; (ii) *x*, −*y*+1, *z*−1/2; (iii) −*x*, *y*, −*z*+1/2; (iv) −*x*, −*y*+1, −*z*.

### Hydrogen-bond geometry (Å, º)

**Table:**

*D*—H···*A*	*D*—H	H···*A*	*D*···*A*	*D*—H···*A*
N1—H1*A*···O1^v^	0.88 (5)	2.00 (5)	2.879 (4)	175 (4)
N1—H1*B*···Br1^vi^	0.88 (4)	2.58 (4)	3.450 (3)	179 (4)
N1—H1*C*···Br1^vii^	0.89 (4)	2.51 (4)	3.321 (3)	152 (3)
O4—H4···O3^ii^	0.83 (2)	2.01 (2)	2.828 (3)	166 (5)
O3—H3*A*···Br1^vi^	0.84 (5)	2.50 (5)	3.335 (3)	170 (4)
O3—H3*B*···Br1^viii^	0.84 (2)	2.55 (3)	3.296 (3)	148 (4)

Symmetry codes: (ii) *x*, −*y*+1, *z*−1/2; (v) −*x*+1/2, −*y*+3/2, *z*+1/2; (vi) *x*, −*y*+2, *z*−1/2; (vii) −*x*+1/2, −*y*+3/2, *z*−1/2; (viii) −*x*, *y*, −*z*+3/2.
